# Antioxidant Activities *in vitro* of Water and Liposoluble Extracts Obtained by Different Species of Edible Insects and Invertebrates

**DOI:** 10.3389/fnut.2019.00106

**Published:** 2019-07-15

**Authors:** Carla Di Mattia, Natalia Battista, Giampiero Sacchetti, Mauro Serafini

**Affiliations:** Faculty of Biosciences and Technologies for Agriculture, Food, and Environment, University of Teramo, Teramo, Italy

**Keywords:** entomophagy, edible insects, edible invertebrates, non-enzymatic antioxidant capacity, novel foods, dietary habits, sustainable nutrition

## Abstract

A new global interest in entomophagy, the practice of eating insects, and invertebrates, arise from the impellent necessity of preserving agriculture resources and to obtain a drastic reduction of the ecological impact of animal food on the planet. The composite nutritional content, direct consequences of a plant-based feeding, associated with the undoubtedly ecological properties, suggest for insects a role as sustainable and functional foods. We aim to investigate the ability of water and liposoluble extracts, obtained by 12 commercially available edible insects and two invertebrates, to display an antioxidant effect *in vitro*. Results show that water-soluble extracts of grasshoppers, silkworm, and crickets display the highest values of antioxidant capacity (TEAC), 5-fold higher than fresh orange juice, while evening cicada, giant water bugs, Thai zebra tarantula, and black scorpions have negligible values. Grasshoppers, African caterpillars, and crickets have the highest levels of reducing power (FRAP), double than fresh orange juice. Grasshoppers, black ants, and mealworms contain the highest levels of total polyphenols, while Thai zebra tarantula, black scorpions, and giant water bugs are positioned at the bottom of the ranking. The liposoluble fraction of silkworm, evening cicada, and African caterpillars shows highest level of TEAC, twice than olive oil, while Thai zebra tarantula, palm worm, and black ants are placed at the bottom of the ranking. Edible insects and invertebrates represent a potential source of antioxidant ingredients with an efficiency related to their taxonomy and eating habits. More evidences are needed in order to understand if the practice of eating insects and invertebrates might contribute to modulate oxidative stress in humans.

## Introduction

Entomophagy, the practice of eating insects and invertebrates, has accompanied the human history through the centuries, playing a significant role in cultural and religious practices (1–3). According to the Food and Agriculture Organization of the United Nations (FAO), insects are part of the common diet of at least two billion people in the world (4). Among the different species available for human consumption, *Coleoptera* (beetles), *Lepidoptera* (African caterpillars), and *Hymenoptera* (bees, wasps and ants) represent 31, 18, and 14% of total insect consumption (4). However, in most western countries, the habit of eating insects is often considered a primitive practice inducing disgust on the basis of insect morphologies.

Recently, a new global interest in edible insects and invertebrates arise from the impellent necessity of preserving agriculture resources to feed the nine billion world's population predicted for 2050 and to obtain a drastic reduction of the ecological impact of meat and derivatives on the planet (5, 6). In this view, livestock production is responsible of about 14.5% of total human-induced greenhouse gas emissions (GHG) (7), one of the main factors inducing climate changes. Under an ecological point of view, insects represent a highly sustainable replacement of meat and animal products, they produce enormously less amount of GHG and require less water, land, and feed to develop their life cycle, compared to livestock (4). Moreover, under a nutritional point of view, insects and invertebrates represent a good source of bioavailable high-quality proteins and essential amino acids, polyunsaturated fatty acids, minerals such as iron, zinc, and potassium, B vitamins, and insoluble fiber, as chitin (4).

As overall, their composite nutritional quality, direct consequences of a plant-based feeding, associated with the undoubtedly ecological properties, suggest for insects a conceivable role as sustainable and functional foods. However, despite insects have been used in traditional medicine in wound healing and as curative therapy for respiratory disorders and stomachache (8, 9), there is scarce evidences about functional properties of insects and invertebrates in the literature. Recently, 2 weeks of consumption of a breakfast enriched with 25 g of cricket powder, was shown to reduce circulating levels of the pro-inflammatory cytokine TNF-α in plasma of 20 healthy adults (10). Moreover, hydrolysates from *Tenebrio molitor* larvae displayed free radical scavenging activity (11, 12) and peptides fraction from the locust *Schistocerca Gregaria* exhibit inhibitory activity of lipoxygenase and cyclooxygenase2 (12). In myoblast cell lines, the cricket *Brachytrupes orientalis* has been shown to decrease intracellular reactive oxygen species (ROS) production, lipid peroxidation, and up-regulating expression of protein Nrf2 and glutathione S-transferase, involved in the red-ox response to stress, following high glucose stimulation (13). However, no evidence is present in the literature about a screening of the antioxidant properties of insects and invertebrates available for human consumption.

In this work, we aim to investigate and compare the ability of water and liposoluble extracts, obtained by commercially available edible insects and invertebrates, representative of different species and eating habits, to exhibit an antioxidant effect *in vitro*.

## Materials and Methods

### Sample Preparation

Edible insects and invertebrates, purchased from Eatgrub (UK) and Insectemente-votre (FR), are described in Table 1 according to name, family classification, dietary habits, and energy content. Orange juice was freshly made by oranges purchased in local supermarkets. Olive oil was purchased in local supermarket. The water content of the insects was within the range 2–7%, with the exception of black ants (46%) and black scorpions (22%), whilst the water content of orange juice was 88%. Before samples size reduction, wings, and paws in grasshoppers and stingers in scorpions were removed and discharged according to supplier instructions; grinding of the samples was carried out in a lab-scale mixer (Bimby TM31, Vorwerk, Wuppertal, Germany). All the other samples were grounded without any pre-treatment since, as stated in the labels, they were ready to use.

**Table 1:** Classification, dietary habits, and energy values of the selected edible insects and invertebrates.

**Order**	**Name**	**Family**	**Dietary habits**	**Energy value (kcal/100 g)**
**Insects**				
Coleoptera	Mealworms (*Tenebrio molitor*)	Tenebrionidae	Herbivorous	550
Coleoptera	Buffalo worms (*Alphitobius diaperinus*)	Tenebrionidae	Herbivorous	484
Coleoptera	Palm worm larvae (*Rhynchophorus ferrugineus*)	Curculionidae	Herbivorous	n.a.
Hemiptera	Evening cicada (*Tanna japonensis*)	Cicadidae	Plant vascular system	n.a.
Hymenoptera	Black ants (*Lasius niger*)	Formicidae	Herbivorous and saprophagous	329
Lepidoptera	African caterpillars (*Imbrasia oyemensis)*		Herbivorous	n.a.
Lepidoptera	Silkworm (*Bombyx mori*)	Bombycidae	Herbivorous (mulberry leaves)	487
Orthoptera	Grasshoppers (*Calliptamus italicus*)	Acrididae	Herbivorous (leguminous, alfalfa)	559
Orthoptera	Crickets (*Acheta domesticus*)	Gryllidae	Herbivorous	458
Orthoptera	Mini crickets (A*cheta domesticus*)	Gryllidae	Herbivorous	487
Rhynchota	Giant water bugs (*Lethocerus indicus*)	Belostomatidae	Carnivorous	n.a.
Scolopendromorpha	Scolopendra gigantea (*Scolopendra*)	Scolopendridae	Herbivorous	n.a.
**Invertebrates**				
Arachnida	Thai zebra tarantula (*Haplopelma albostriatum*)	Theraphosidae	Carnivorous	450
Scorpiones	Black scorpion (*Pandinus imperator*)	Scorpioniade	Carnivorous	n.a.

### Sample Defatting and Extraction of the Lipo-Soluble Fraction

Grounded insects (4 g) were defatted with three cycles of hexane washing (25 mL). Each time, the mixture was first vortexed (1 min) and then centrifuged at 2,346 g in an ALC 4237R centrifuge (Milan, Italy) for 10 min; at each cycle the surnatant was recovered and the three combined fractions represented the liposoluble extracts. All the extracts were concentrated at 37°C under vacuum using a rotative evaporator (Büchi Labortechnik, Flawil, Switzerland). The lipid-free solids were air-dried at room temperature until complete removal of hexane and then used for the extraction of the water-soluble extracts.

### Extraction of the Water-Soluble Fraction

Dried and defatted insects (1 g) were added to 25 mL of distilled water and homogenized for 1 min using a Vortex mixer (IKA, Staufen, Germany). The homogenate was put in a 50 mL capped vial, wrapped in an aluminum sheet and extracted for 1 h in a shaker, which was kept at 18°C under dark conditions. The homogenate was centrifuged for 15 min at 2,346 g in an 4237R ALC (Milan, Italy) centrifuge set at 4°C and the surnatant was filtered through cellulose filters; water was added to a final volume of 25 mL. The extract obtained was then used to quantify the total polyphenols index (TPI) and to determine the antiradical and reducing activity by the ABTS and FRAP methods, respectively.

### Radical Scavenging Activity (TEAC)

The radical scavenging activity was measured by the ABTS [2,2′-azino-nis (3-ethylbenzthiazoline-6-sulfuric acid)] radical cation decoloration assay, as described by Re et al. (14). The bleaching rate of ABTS^+•^ in the presence of the sample was monitored at 734 nm using a Perkin Elmer (Boston, MA, USA) Lambda Bio 20 spectrophotometer. The ABTS^+•^ stock solution was diluted either in water or ethanol up to an Abs of 0.70 ± 0.02 for the analysis of the aqueous and apolar extracts, respectively. Volumes of 2.97 and 2.88 μL of ABTS^+•^ solution were used for hydrophilic and lipophilic extracts, respectively. The reaction was started by the addition of 30 and 120 μL of hydrophilic and lipophilic extracts to aqueous and ethanolic ABTS solution, respectively. The ABTS^+•^ bleaching was monitored at 30°C and the decoloration after 5 min was used as the measure of antioxidant activity. Radical scavenging activity was measured as Trolox Equivalents Antioxidant Capacity (mmol of Trolox eq. per 100 g of sample) and calculated by the ratio of the correlation coefficient of the dose–response curve of the sample and the correlation coefficient of the dose–response curve of the standard compound. Trolox was used as standard for the calculation of the radical scavenging activity for both the water soluble (TEAC_aq_) and liposoluble extracts (TEAC_lipo_).

### Ferric Reducing Antioxidant Power (FRAP)

The reducing activity of the samples was determined according to the method described by Benzie and Strain (15) with some modifications. One hundred microliters of opportunely diluted sample extract were added to 2,900 μL of the FRAP reagent obtained by mixing acetate buffer (300 mM, pH 3.6), TPTZ (2,4,6-tripyridyl-s-triazine) 10 mM solubilized in HCl 40 mM and FeCl_3_ 20 mM in the ratio 10:1:1. The absorbance change was followed at 593 nm for 6 min. A calibration plot based on FeSO_4_·7H_2_O was used and results were expressed as mmoles of Fe^2+^ per 100 grams of defatted weight.

### Total Polyphenols Index (TPI)

The TPI was determined according to a procedure modified from Giacintucci et al. (16). The sample extract (0.1 mL) was diluted with deionized water to a volume of 5 mL and then 500 μL of Folin–Ciocalteu reagent was added; after 3 min, 1.5 mL of a 25% Na_2_CO_3_ solution was added and then deionized water up to 10 mL final volume. Solutions were maintained at room temperature under dark conditions for 60 min and the total polyphenols content was determined at 765 nm using a Perkin Elmer Lambda Bio 20 spectrophotometer. Gallic acid standard (Fluka, Buchs, CH) solutions were used for calibration purposes. Results were expressed as milligrams of gallic acid equivalents (GAE) per 100 g of sample.

## Results

**Figure 1:**
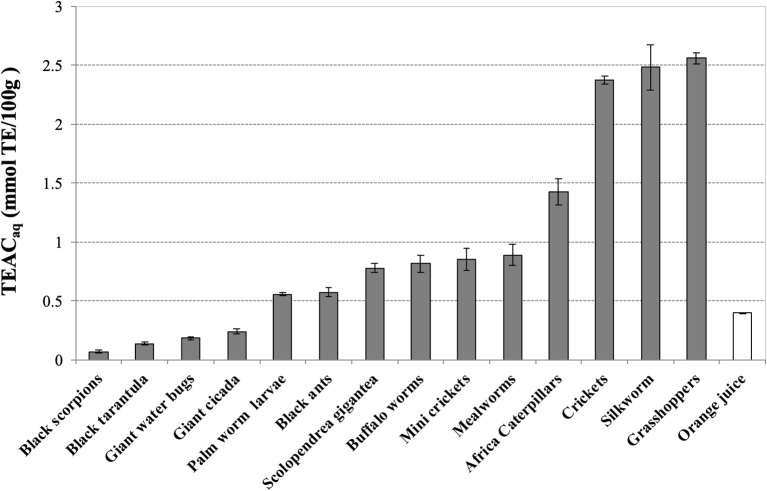
Trolox Equivalent Antioxidant Capacity (TEAC) of water-soluble extracts of edible insects, invertebrates and fresh orange juice. Values are expressed as TEAC_aq_ (mmolTE/100 g of defatted sample or 100 mL in the case of orange juice) and are the mean ± SD in triplicate. TE, Trolox Equivalents.

The water-soluble extracts of grasshoppers (2.55 ± 0.05), silkworm (2.48 ± 0.19), and crickets (2.37 ± 0.03) display the highest values of antioxidant capacity, measured as TEAC_aq_ (mmol TE/100 g), 5-fold higher than fresh orange juice (0.40 ± 0.01), as described in Figure 1. In the second group of antioxidant activity we found African caterpillars (1.43 ± 0.11), mealworms (0.89 ± 0.09), mini crickets (0.85 ± 0.09), buffalo worms (0.82 ± 0.07), scolopendra (0.78 ± 0.04), black ants (0.57 ± 0.04), and palm worm (0.55 ± 0.01). As overall, majority of extracts, except for evening cicada (0.24 ± 0.02), giant water bugs (0.18 ± 0.01), Thai zebra tarantula (0.13 ± 0.01), and black scorpions (0.06 ± 0.01), have level of antioxidant capacity higher than fresh orange juice.

**Figure 2:**
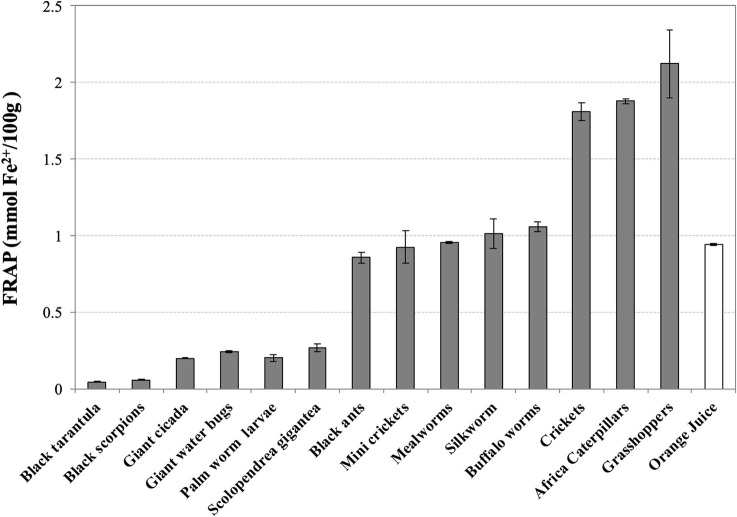
Ferric Reducing Antioxidant Potential (FRAP) of water-soluble extracts of edible insects, invertebrates, and fresh orange juice. Values are expressed as FRAP (mmol Fe^2+^/100 g of defatted sample or 100 mL in the case of orange juice) and are the mean ± SD in triplicate.

For what concerns the values of reducing power of water-soluble extracts, grasshoppers (2.12 ± 0.22), African caterpillars (1.88 ± 0.02), and cricket (1.81 ± 0.06) display the highest FRAP values (mmol Fe^2+^/100 g), about 2-fold higher than orange juice (0.94 ± 0.01) (Figure 2). Black ants, mini crickets, mealworms, silkworms, and buffalo worms are in intermediate position with values comparable to orange juice. Thai zebra tarantula (0.04 ± 0.00) and black scorpions (0.06 ± 0.00) have extremely low values of FRAP.

**Figure 3:**
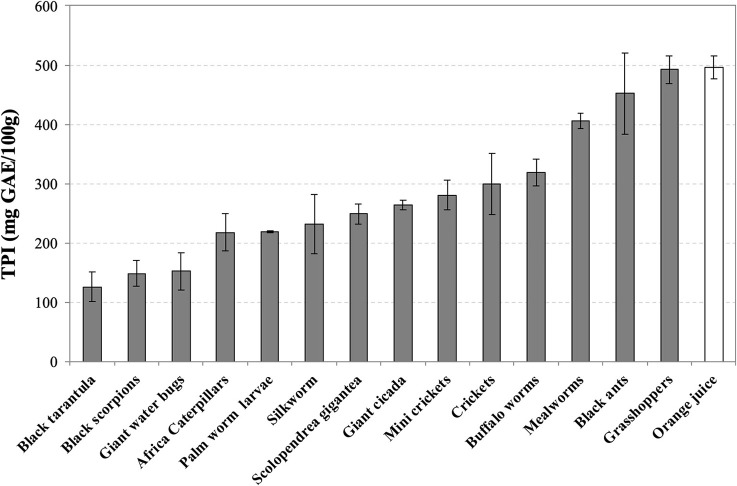
Total Polyphenol Index (TPI) of water-soluble extracts of edible insects, invertebrates, and fresh orange juice. Values are expressed as mgGAE/100 g of defatted sample or 100 mL in the case of orange juice and are the mean ± SD in triplicate. GAE, Gallic acid equivalent.

Grasshoppers (492 ± 24), black ants (452 ± 68), and mealworms (406 ± 12) contain the highest levels of TPI (mg GAE/100 g), followed by buffalo worms (319 ± 22) and crickets (299 ± 51) (Figure 3). As for the TEAC values, Thai zebra tarantula (126 ± 25), black scorpions (148 ± 21), and giant water bugs (152 ± 31) are positioned at the end of the ranking. Grasshoppers (492 ± 24) and black ants (452 ± 68) are the only insects with comparable levels of TPI respect to orange juice (496 ± 19).

**Figure 4:**
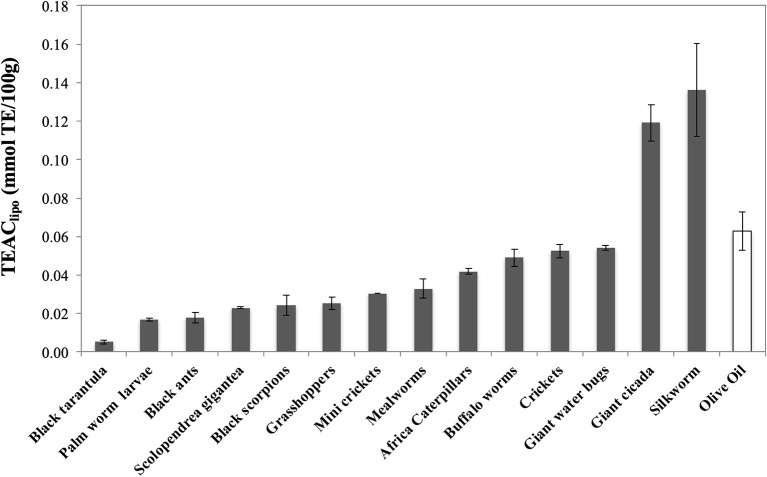
Trolox Equivalent Antioxidant Capacity (TEAC) of lipo-soluble extracts of edible insects, invertebrates, and olive oil. Values are expressed as TEAC_lip_ (mmolTE/100 g) and are the mean ± SD in triplicate. TE, Trolox Equivalents.

When the lipophilic fraction of the extracts was tested, silkworm (0.136 ± 0.024) and evening cicada (0.119 ± 0.010) display the highest values of TEAC_lipo_ (mmol TE/100 g), twice than olive oil (0.063 ± 0.010) (Figure 4). As overall, all the other extracts display similar values, with Thai zebra tarantula (0.005 ± 0.001), palm worm (0.017 ± 0.001), and black ants (0.018 ± 0.003) at the bottom of the ranking.

## Discussion

Insects and invertebrates are often consumed as whole and, despite some people still view entomophagy as a disgusting practice, it is becoming a popular trend of human nutrition in European countries. It is already well-known that insects and invertebrates represent a valid source of noble proteins, minerals, vitamins, and fatty acids at low ecological impact, however scarce information are available on their role as source of bioactive ingredients. In this work we have shown that commercially available edible insects and invertebrates represent a potential source of antioxidant ingredients, with an efficiency related to their taxonomy and eating habits.

Consumption of foods rich in antioxidants, such as fruit and vegetables, play an important role in the prevention of oxidative stress-related diseases such as cardiovascular disease, diabetes and cancer (17). The *in vivo* efficiency of antioxidant-rich food is highly dependent from bioavailability and by the presence of an ongoing oxidative stress (18–20). However, a high content of antioxidant in the food matrix it is a primary requisite for a first screening of antioxidant potentiality of novel foods. Since it is always difficult to define if the antioxidant efficiency *in vitro* is high or low, we have compared the results of some edible insects and invertebrates with the values of antioxidant capacity from fresh orange juice and olive oil, respectively, for the water and lipo-soluble fractions of extracts. Crickets, grasshoppers, silkworm, African caterpillars, and evening cicada display values of antioxidant capacity 2- or 3-fold higher than orange juice or olive oil, functional foods that are known to modulate antioxidant network in humans (21, 22). However, despite the promising results *in vitro*, tailored intervention studies are needed to clarify the antioxidant role of edible insects and invertebrates in human.

The phenolic content of our samples, as determined by TPI, does not show higher values for edible insects compared to fresh orange juice, except for grasshoppers, suggesting that their antioxidant capacity is not only due to this class of compounds. It is possible that, as showed previously by other authors (12, 23, 24), proteins might contribute to their antioxidant capacity. However, if we consider that FRAP assay do not measure antioxidant contribution from protein groups, we have remarkable higher values compared to orange juice. Moreover, the main antioxidant compounds in orange juice are ascorbic acid and water soluble phenolics, and for olive oil, tocopherols, and amphiphilic phenolics, our results suggest that edible insects are endowed with a peculiar pattern of redox ingredients, ranging from phenolics, proteins as well as unidentified components, able to counteract oxidative stress from water and lipophilic environment.

Our results show that the antioxidant pattern of the edible insects and invertebrates is different according to taxonomy and dietary habits. As regards the water-soluble extracts, we observe that grasshoppers and crickets (orthoptera) and African caterpillars and silkworm (lepidoptera), all characterized by vegetarian dietary habit, are endowed with the highest antioxidant capacity. On the contrary, carnivorous Thai zebra tarantula (arachnids), black scorpions (scorpions), and giant water bugs (rhynchota), are at lowest position of the ranking. However, the scenario change when we analyzed the antioxidant activity of the liposoluble extracts, where silkworm (lepidoptera) is still at the top of the ranking but followed by the carnivorous giant water bugs (rhynchota) and evening cicada (hemiptera), insects that did not display a significant antioxidant activity in the water soluble fraction.

Our results shows that edible insects and invertebrates are an optimal source of bioactive ingredients and of high quality protein, minerals, vitamins, and fatty acids (4), together with a low environmental impact, highlighting their importance as sustainable novel foods under a nutritional, functional, and ecological point of view. In the view of the raising interest of scientific community, media, and consumer for entomophagy, findings of our study are important also under a public health perspective, providing the basis to develop scientific-based campaigns to promote entomophagy and increasing public awareness about the importance of reducing consumption of food at high ecological impact with edible insects, maintaining or even improving nutritional and functional benefits.

In the future we might adapt specific dietary regimen for insects rearing in order to increase antioxidant content and optimizing nutritional intake for a sustainable and functional animal or human consumption, in agreement with the “one health” concept.

## Conclusion

Edible insects and invertebrates represent a potential source of unexplored redox ingredients with low ecological impact, with an antioxidant efficiency related to their taxonomy and eating habits. More evidence is needed in order to understand if the practice of eating insects and invertebrates might contribute to modulate oxidative stress in humans and the identification of their bioactive ingredients.

## Data Availability

The datasets generated for this study are available on request to the corresponding author.

## Author Contributions

MS designed the experiment. CD conducted the experiments. MS, NB, GS, and CD contributed to data analysis and interpretation and contributed to the manuscript drafting.

### Conflict of Interest Statement

The authors declare that the research was conducted in the absence of any commercial or financial relationships that could be construed as a potential conflict of interest.

## Abbreviations

FRAP: ferric reducing antioxidant power
GAE: gallic acid equivalent
ROS: reactive oxygen species
TEAC: Trolox equivalent antioxidant capacity
TPI: total polyphenol index
GHG: greenhouse gas emissions
CVD: cardiovascular disease.
